# Shedding New Light on Cancer Metabolism: A Metabolic Tightrope Between Life and Death

**DOI:** 10.3389/fonc.2020.00409

**Published:** 2020-03-31

**Authors:** Matthias Läsche, Günter Emons, Carsten Gründker

**Affiliations:** Department of Gynecology and Obstetrics, University Medicine Göttingen, Göttingen, Germany

**Keywords:** cancer, metabolism, microenvironment, metastasis, therapy

## Abstract

Since the earliest findings of Otto Warburg, who discovered the first metabolic differences between lactate production of cancer cells and non-malignant tissues in the 1920s, much time has passed. He explained the increased lactate levels with dysfunctional mitochondria and aerobic glycolysis despite adequate oxygenation. Meanwhile, we came to know that mitochondria remain instead functional in cancer cells; hence, metabolic drift, rather than being linked to dysfunctional mitochondria, was found to be an active act of direct response of cancer cells to cell proliferation and survival signals. This metabolic drift begins with the use of sugars and the full oxidative phosphorylation via the mitochondrial respiratory chain to form CO_2_, and it then leads to the formation of lactic acid via partial oxidation. In addition to oncogene-driven metabolic reprogramming, the oncometabolites themselves alter cell signaling and are responsible for differentiation and metastasis of cancer cells. The aberrant metabolism is now considered a major characteristic of cancer within the past 15 years. However, the proliferating anabolic growth of a tumor and its spread to distal sites of the body is not explainable by altered glucose metabolism alone. Since a tumor consists of malignant cells and its tumor microenvironment, it was important for us to understand the bilateral interactions between the primary tumor and its microenvironment and the processes underlying its successful metastasis. We here describe the main metabolic pathways and their implications in tumor progression and metastasis. We also portray that metabolic flexibility determines the fate of the cancer cell and ultimately the patient. This flexibility must be taken into account when deciding on a therapy, since singular cancer therapies only shift the metabolism to a different alternative path and create resistance to the medication used. As with Otto Warburg in his days, we primarily focused on the metabolism of mitochondria when dealing with this scientific question.

## Metabolism in Cancer

The metabolic plasticity and context-dependent diversity of its phenotype characterize cancer and its development into tumors and metastases. In this context, six hallmarks of cancer metabolism have been described (1, 2), which include: (1) increased uptake of glucose and amino acids from tumor microenvironment and concomitant delivery of lactate and protons to tumor cells; (2) increase of the glycolysis pathway, the pentose phosphate pathway (PPP), and the tricarboxylic acid (TCA) cycle intermediates to build and sustain the aberrant proliferation of cancer cells; (3) a more mechanical uptake of nutrients via phagocytosis, entosis, and micro- and macropinocytosis; (4) increased need and utility of nitrogen derivatives and their conversion to nucleotides (pyrimidines, purines), non-essential amino acids, and polyamines; (5) changes in metabolite-driven gene regulation by, for instance, methylation, acetylation and succinylation; and (6) bilateral metabolic interaction through the exchange of nutrients and amino acids or the influence of growth factors or environmental conditions, such as hypoxia or redox stress on the microenvironment or their influence on metabolism and the different signaling levels of cancer cells. Because of the complexity, the cancer metabolism characteristics mentioned above occur in varying degrees and contexts of many different types of cancer; therefore, they can be only roughly defined and are not conclusive in their number or form.

Nearly 100 years ago, Otto Warburg (3–5) discovered the first metabolic differences between normal and cancer cells. Since then, the knowledge of the correlations of metabolism of cancer cells associated with their environmental healthy tissue and the impact of these correlations on the degree of cell tumor growth and metastatic spreading has grown constantly and has adopted an unimagined complex image over the last 15 years. Warburg and his following scientific colleagues started from the now refuted assumption that the change in glucose consumption and the aggravated increase in acidifying the tumor-microenvironment were due to irreversible mitochondrial damage (4). Today we know that cancer cells, due to the requirements for exaggerated proliferation and the resulting increased need for metabolites, actively put forward advancing the path of glycolysis, usually associated with the pathway of oxidative citrate cycle catabolism (6). However, why, all of a sudden, do cancer cells “change their mind”? Why are they using aerobic glycolysis, and why are they not also using the citric acid cycle, given that the latter, when compared to glycolysis, which produces only two molecules of ATP per molecule of glucose, results in 36 or 38 molecules of ATP and GTP, respectively through the electron transport chain (ETC)?

**Figure 1:**
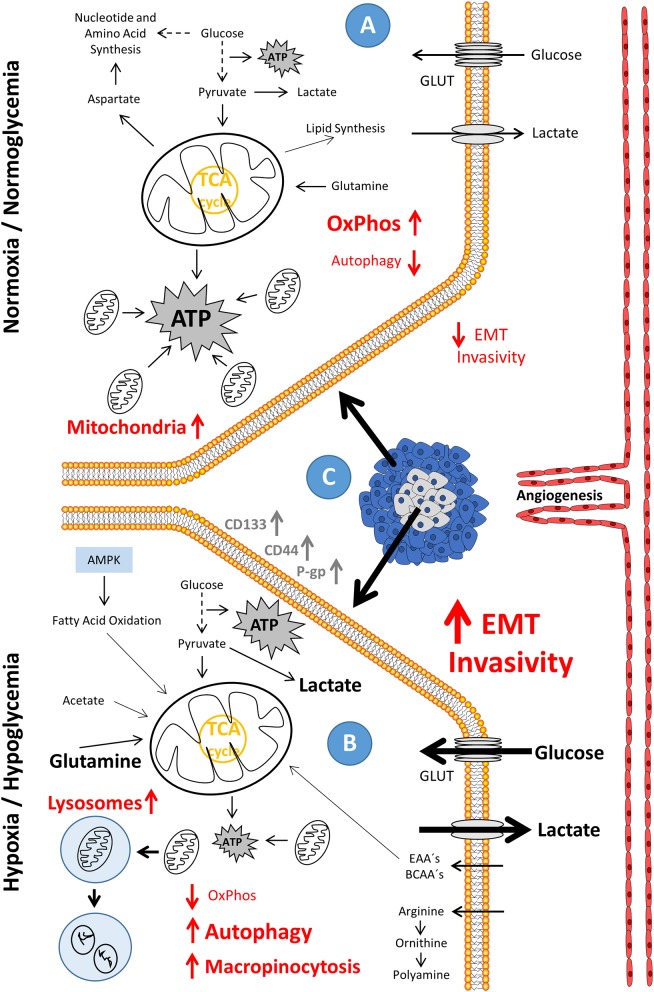
Phenotypic evolution in hypoxia/hypoglycemia-exposed metastatic cancer and the accompanying drift in energy metabolism. **(A)** In the state of normoxia/normoglycemia, the condition that resembles the environment in tumors close to the vascular system and lined by outer proliferating tissue **(C)**, ATP is produced through the commonly used energy metabolism paths, with the major part of ATP supplied by oxidative phosphorylation (OxPhos). Enzymes, which are related to metastasis are low expressed; and there is only a small tendency of cells to invade the surrounding tissue. **(B)** In the state of hypoxia/hypoglycemia, the condition which resembles the environment in tumors distant to the vascular system and surrounded by resting tissue **(C)**, oxidative phosphorylation (OxPhos), reduced by a lower protein content (possibly induced as a result of a strong downregulation of transcription), is greatly diminished and autophagy fully activated. ATP is predominantly produced through glycolysis. Enzymes, which are related to metastasis, show a high expression level; and there is a high tendency to invade the surrounding tissue. AMPK, AMP-activated protein kinase; ATP, adenosine triphosphate; BCAA's, branched-chain amino acids; EAA's, essential amino acids; EMT, epithelial-mesenchymal transition; GLUT's, glucose transporters; OxPhos, oxidative phosphorylation; TCA, tricarboxylic acid cycle.

**Figure 2:**
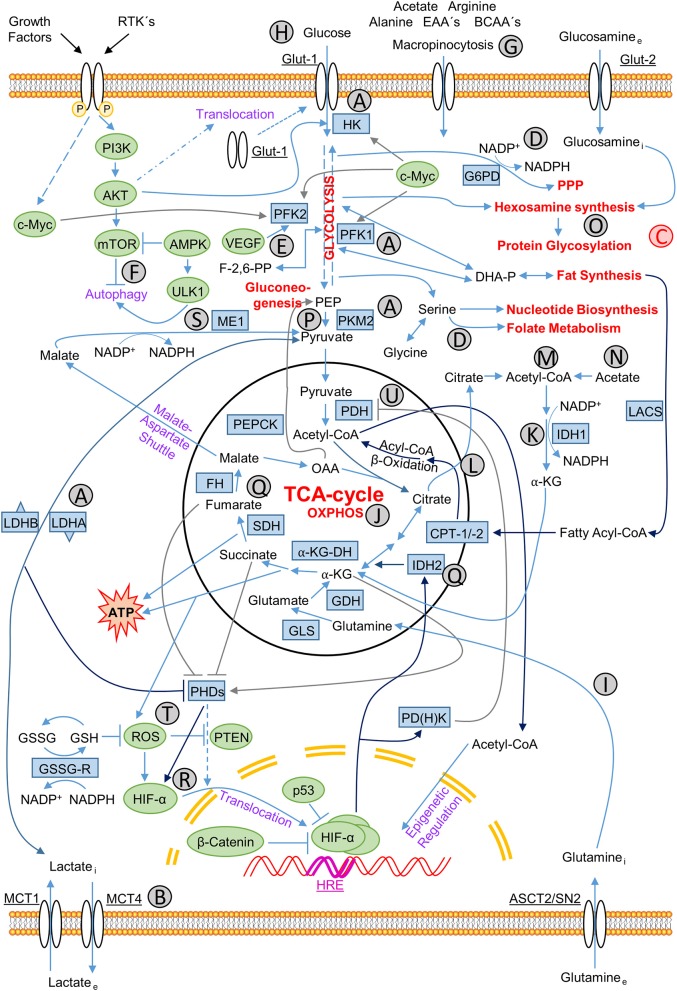
Metabolic-regulatory relationships in cancer cells. Relationships between influxes and effluents of metabolites in cancer cells, individual metabolic pathways of energy production and production of precursor molecules for biosynthesis of lipids, nucleotides, and macromolecules for membranes and organelles, NADPH for production of reducing equivalents and the control of all these interactions by appropriate signaling pathways. Signaling molecules are marked green, participating enzymes blue and main catabolic and anabolic pathways red. Signaling molecules: AKT, protein kinase B; AMPK, AMP-activated protein kinase; β-catenin, cadherin-associated protein; c-Myc, MYC; HIF-1α, hypoxia-inducible factor, subunit 1α; mTOR, Mammalian target of rapamycin; p53, tumor suppressor p53; PI3K, phosphoinositide 3-kinase; PTEN: phosphatase and tensin homolog; ROS: reactive oxygen species; ULK1, Unc-51-like autophagy-activating kinase; VEGF: vascular endothelial growth factor. Metabolic enzymes: α-KG-DH, α-ketoglutarate dehydrogenase; CPT-1/2, carnitine palmitoyltransferase 1/2; FH, fumarate dehydrogenase; G6PD(H), glucose-6-phosphate dehydrogenase; GDH, glutamate dehydrogenase; GLS, glutaminase; GSH, glutathione (reduced form); GSSG, glutathione (oxidized form); GSSG-R, GSSG-reductase; HK, hexokinase; IDH1/2, isocitrate dehydrogenase 1/2; LACS, long-chain acyl-coenzyme A (CoA) synthetase; LDHA/B, lactate dehydrogenase A/B; ME1, malic enzyme 1; NADP^+^/NADPH, nicotinamide adenine dinucleotide phosphate (oxidized/reduced form); PDH, pyruvate dehydrogenase; PD(H)K, pyruvate dehydrogenase kinase; PEPCK, phosphoenolpyruvate carboxykinase [GTP-dependent]; PFK1/2, phosphofructokinase 1/2; PHDs, Prolyl 4-hydroxylase dioxygenases; PKM2, pyruvate kinase type M2; SDH, succinate dehydrogenase. Transporter proteins: ASCT2/SN2, glutamine transporter; Glut-1, glucose transporter type 1, also erythrocyte/brain hexose facilitator (gene: SLC2A1); Glut-2, glucose transporter type 2 (gene: SLC2A2); MCT1/4, monocarboxylate transporter 1/4 (gene: SLC16A1/3). Metabolites: α-KG, α-ketoglutarate; BCAA's, branched-chain amino acids; EAA's, essential amino acids; OAA, oxaloacetate; PEP, phosphoenolpyruvate. Others: HRE, hypoxia-response-element. Characters A–U: **(A)** glycolytic enzymes up-regulated by hypoxia; **(B)** lactate efflux; **(C)** synthesis pathways branched off from glycolysis path; **(D)** NADPH producing pathways; **(E)** PFK2 activity stimulated by VEGF; **(F)** autophagy regulation; **(G)** extracellular uptake of nutrients via macropinocytosis; **(H)** glucose uptake; **(I)** glutamine metabolism; **(J)** oxidative phosphorylation; **(K)** reductive NADPH production; **(L)** citrate as central metabolite; **(M)** citrate reduction to acetyl-CoA; **(N)** acetate derived acetyl-CoA synthesis; **(O)** protein glycosylation via hexosamine synthesis; **(P)** glycolysis flux controlling PKM2 activity; **(Q)** often mutated enzymes and the resulting oncometabolites in the TCA cycle; **(R)** stabilization of HIF-α; **(S)** NADPH production through oxidative decarboxylation catalyzed by ME1; **(T)** ROS-mediated PTEN inactivation and HIF-α stabilization; **(U)** suppression of PDH via PDHK leading to the shift from OXPHOS to glycolysis.

One explanation is given by the fact that it is wrong, unlike Otto Warburg understood, that cancer depends on aerobic glycolysis only as it, next to glycolysis, tread the oxidative path of mitochondria across ETC for about 5–15% of the glucose flow. On the one hand, it is correct to say that neoplasia can arise as a cause of dysfunctional mitochondria; otherwise, this means not that oncogenic development is solely glycolysis-dependent (1); rather, the metabolic drift to aerobic glycolysis is due to the rapidly proliferating neoplasia meeting its metabolic limits. Hypoxia increases in the core of the developing tumor (Figure 1). Hypoxia up-regulates the transcription of target genes related to the hypoxia response element (HRE) (like genes coding for erythropoiesis, glycolysis, and angiogenesis) through the stabilized hypoxia-inducible factor 1-alpha (HIF-1α), translocating to the nucleus and binding HIF-1β to an active complex. This occurs via the cell's oxygen sensor, the hypoxia-inducible prolyl hydroxylase protein 2 (PHD2), leading to higher expression levels of glycolytic enzymes which is responsible for the uptake of nutrients, essentially glucose (Figure 2A). Enzymes for the apparent “waste” product lactate—the lactate transporter proteins MCTs, which accelerate the release of the glycolysis product (7, 8)—show higher expression levels as well (Figure 2B). These enzymes include, for instance, hexokinase 2 (HK2), phosphofructokinase 1 (PFK1), aldolase A (ALDOA), phosphoglycerate kinase 1 (PGK1), pyruvate kinase (PK), and lactate dehydrogenase A (LDH-A). In addition, tumorigenesis shows a much higher utilization of different metabolites next to glucose, which is due to the strong cell growth and division. Augmented aerobic glycolysis gives the developing tumor the opportunity to quickly generate the metabolic bricks required for cell assembly and division. Energy, nucleotides (non-essential), and essential amino acids, polyamines, fatty acids, and reductive equivalents (Figure 2C) are supporting cell proliferation and its need for phospholipids for the construction of membranes, mitochondria, lysosomes, and so on. This production works through various ways, which we will discuss in more detail in the later context of this review (9–13).

Furthermore, concerning ATP as an energy carrier, the unfinished glucose oxidation with lactate as the end-product offers a decisive growth benefit. Although glycolysis produces only 6% of the ATP per mol glucose when comparing it to the outcome through the Krebs cycle and ETC, it is about two orders of magnitudes faster as a result of the much faster chemical reaction (14, 15). While not efficiently enough in utilizing the sugar, it is quite worthy for the aberrant proliferation, and it produces fast energy.

Glycolysis and the resulting metabolism pathways, like pentose phosphate pathway (PPP) or one-carbon (folate) cycle (Figure 2D), offer the benefit of producing sufficient reductive forces with NAD(P)H and glutathione to neutralize reactive oxygen species (ROS). ROS are, for example, the hyperoxide anion (O2·^−^), the hydroxyl radical (HO·), or the hydrogen peroxide (H_2_O_2_), resulting from the rapid metabolism, which would, if not eliminated, depolarize the mitochondrial membrane and lead to apoptosis. However, these same ROS are critically important in the initiation phase of tumorigenesis as they provide the somatic or germline mutations, like in the fumarate hydratase (FH), succinate dehydrogenase (SDH), or isocitrate dehydrogenase (IDH1/2) genes, necessary to promote tumorigenesis (16).

Furthermore, glucose uptake regulation, lactate excretion, and increased tumor microenvironment acidification through the monocarboxylate transporters (MCTs) (Figure 2B) also are responsible for tumor survival (17–19). The tumor development is therefore dependent on influencing the microenvironment to provide the needed signaling and metabolic fuel to keep the aberrant metabolism going, contributing to the evasion of the surveilling immune system. Only recently, the tumor microenvironment research has become more attentive to their great value. The influence of the developing tumor on its microenvironment or vice versa is a relationship characterized essentially by the aggressiveness of tumor development and its ability to promote metastasis. For the tumor to make its way through the body and to proliferate without being affected by the immune system, it is crucial for it to be capable of further growth despite adverse conditions in its development, such as increasing hypoxia, glucose deprivation, lack of amino and fatty acids, and fatal hyper-acidification. As research has recently revealed, cancer cells, in their entire development, always move on a narrow metabolic ridge between life and death, which is crucial for a patient's survival.

Depleted from oxygen and glucose supply during its growth and with the diffusion coefficient of glucose being far greater than that of oxygen, the inner core of the developing tumor gets increasingly hypoxic and hypoglycemic. Essential nutrients, too, like glutamine, important for the nitrogen containing molecules derived from them, significantly decrease in the nutrient balance (Figure 1B). However, the tumor escapes these limiting conditions by stimulating the microenvironment to develop the so-called tumor micro-vascularization or neo-vascularization. The endothelial development needs the stimulus of the vascular endothelial growth factor (VEGF) to form and diversify new blood vessels that grow toward the tumor and provide it with the necessary building materials (Figure 1C). Induction by an important enzyme of the energy metabolism, the phosphofructokinase-2/fructose-2,6-bisphosphatase 3 (PFKFB3) that catalyzes phosphorylation of fructose-6-phosphate to form fructose-2,6-bisphosphate stimulated by VEGF (Figure 2E), drives angiogenesis and the migration of the endothelia by regulating the vasculature of stress fibers (20, 21). Increasing lactate secretion by enhanced glycolysis promotes angiogenesis through HIF1α activation and VEGF receptor 2 (VEGFR-2) up-regulation (22).

Lactic acidosis, a high lactate concentration at acidic pH, rescues the cancerous cells from cell death threatened by glucose withdrawal (18). No glucose means no glycolysis and no PPP. Fatty and amino acids sustain energy metabolism, but they are not able to sustain PPP, essential for tumor survival. Cancer cells change to a sleeping mode. Maybe it comes to a G0/G1 phase arrest, possibly via the up-regulation and stabilization of the G1/S transition inhibitor p27 and the down-regulation and destabilization of Skp2. Skp2 is a member of the SCF complex. P27 recognizes it, and after it is poly-ubiquitinated and proteolysed. Thereby, lactic acidosis activates autophagy [(18, 23, 24); Figures 1B, 2F]. Autophagy, namely the breakdown and utilization of cell-derived proteins and organelles, provides the tumor with metabolites for survival in deprived glucose conditions. If re-vascularization of the tumor is successful, the cells of the tumor bud, preceding the proliferative tip of the tumor, may involve a transition to increased migration and invasion, assuming mesenchymal structures initiating the so-called “metastatic cascade” or epithelial-mesenchymal transition (EMT).

The resulting phenotypic and metabolic plasticity of developing malignancies requires functional interaction with the non-malignant constituents of the microenvironment of the tumor. Among others, cancer stem cells (CSCs) exist with a self-renewing potential responsible for the local progression and resurgence of the tumor. They show, except for those of ovarian tumors, a predominantly glycolytic metabolism. The microenvironment of the tumor—specifically, that of the so-called tumor-associated fibroblasts (TAFs)—is reprogrammed for glycolysis and thus support MCT-4 transporter-driven lactate secretion into the tumor milieu and, via the MCT-1 transporter-driven ingestion of lactate, the transport to the oxidative metabolism within the mitochondria of the malignant cells. Many other tumors induce autophagy within the TAFs and thus exploit the generated alanine availability through the extracellular uptake of proteins via so-called macropinocytosis (Figure 2G)—a behavior similar to parasitism. Metabolic competition with immune effector cells for nutrients, with deprived availability, is also one of the reasons for inadequate immune surveillance of tumor progression by immune system effector cells that resemble cancer cells in their metabolism.

Metabolic rewiring promotes tumorigenesis, on the one hand by the increased uptake of glucose (Figures 1A,B, 2H), on the other hand via the glutamine metabolism (Figures 1A,B, 2I). This contributes, in an oxidative manner, to the energy production in the citrate cycle and subsequent ETC (Figures 1A,B, 2J) or, in a reductive way, to fatty acid and cholesterol synthesis via the production of NADPH (Figure 2K), maintaining redox homeostasis (25–28). The metabolically flexible use of other carbon sources, such as lactate, glycine, serine, or acetate, or the flexible change of use of glycolysis, OxPhos, and fatty acid oxidation as an energy source demonstrate the immense plasticity of the response of cancer cells to the ever-changing environmental conditions within their tumor microenvironment (29–33). Reversibility of the TCA cycle and various mitochondrial anaplerotic circuits provide the required adaptation of metabolism (34, 35). Citrate is one of these important central metabolites that is oxidatively converted in TCA as a fuel (Figure 2L) and reductively by transferring an acetyl residue from the citrate to coenzyme A by means of ATP citrate lyase (ACLY) and to acetyl-coenzyme A (acetyl-CoA) (Figure 2M). The enzyme ACLY used here is specific of cancer cells and is not expressed by normal cell proliferation. The acetylating reactions are using acetyl-CoA, regulating transcription and cytoplasmic processes, such as autophagy, in addition to its use in fatty acid and cholesterol synthesis (35–40). α2-macroglobulin (α2M^*^), once activated, for example, signals through the glycoprotein 78 (GRP78) via the tumor and, in addition to activating the AKT pathway, regulates glucose-dependent ACLY and acetate-dependent acetyl-CoA synthetase (ACSS2), the acetyl-CoA synthesis (Figure 2N), and subsequent histone acetylation to induce tumor growth (41).

The hexosamine biosynthesis pathway (HBP) is at the interface of many processes in cancer development. It is very much dependent on the nutritional status of cancer cells, especially the glucose and glutamine supply, as well as other metabolites of other metabolic pathways such as fatty acid (acetyl-CoA) or nucleotide (UTP) metabolism. The HBP branches off from the glycolytic pathway at the level of fructose-6-phosphate, and its metabolic end product uridine diphosphate N-acetylglucosamine (UDP-GlcNAc) mediates many downstream glycosylation steps via its downstream protein O-GlcNAc-transferase (OGT) and the structural changes of the proteins and lipids that are involved in processes that affect cell signaling, gene regulation, and EMT (Figure 2O). The resulting processes are so varied and complex that we refer to Akella et al. (42) for an extensive summary.

The so-called uronic acid or glucuronate pathway branches off from the glycolytic pathway at the level of glucose-6-phosphate via glucose-1-phosphate with its key enzyme—UDP-glucose-6-dehydrogenase (UGDH)—transforming UDP-glucose to UDP-glucuronic acid through activated EGFR signaling. The cancer cell needs UDP-glucuronic acid to produce polysaccharides, like hyaluronic acid—an extracellular matrix component in epithelial tissues. Hyaluronic acid activates cell surface receptors triggering EMT, being responsible for the poor clinical outcome (43, 44). pUGDH reacts with human antigen R (HuR), transforming UDP-glucose (UDP-Glc) to UDP-glucuronic acid (UDP-GlcUA); attenuation of UDP-glucose-derived inhibition of HuR associating with SNAI1 mRNA increases the SNAI1 mRNA-stability. Augmented SNAIL drives epithelial-mesenchymal transition, tumor cell migration and lung cancer metastatic dissemination. Tyrosine 473 phosphorylation of UGDH goes in hand with metastasis and a devastating prognosis for patients with lung cancer (45). Unlike metabolites accumulating because of cancer-causing genetic alterations in metabolic enzymes, normally promoting tumor progression, UDP-Glc is limiting tumorigenesis (46, 47).

Moving from neoplasia, through malignancy to distant metastasis and resting cancer stem cells, the cancer cell re-orients its metabolism and its associated phenotypic de-differentiation several times to survive in the ever-changing microenvironment and maintain steady proliferation or, in the case of cancer stem cells, survive transient senescence to re-metastasize and proliferate in the presence of adequate micro-environmental conditions. With metastatic seeding (dissemination) caused by the epithelial-mesenchymal transition (EMT), the cancer cells gain an increased migratory and invasive potential and associated cytoskeletal modifications for the required motility of the spreading tumor.

The cancer cell regulates evolutionarily highly conserved genes, such as the eukaryotic translation initiation factors 5B or 2 [eIF5B (2)] (48), or the dimeric isoform 2 of pyruvate kinase (PKM2) [(49); Figure 2P], which are widely needed in human embryogenesis. They are obligatory for de-differentiation from the tumor's cell of origin and the reorganization of central carbon metabolism.

## Reprogrammed Metabolism as a Cause of Cancer Development

Neoplasia, or malignant precursor cell, develops from a cell that has lost its normal differentiation character and involvement in the surrounding microenvironment of its host organ. Various factors can lead to cancer.

Research on the biochemical factors, which cause the spreading of the malignancy, has resulted in remarkable progress concerning the relative treatment methods; however, the emergence of resistance unfortunately still poses significant setbacks in therapy. In addition to the surgical methods of curative therapy, resection, transplantation, and ablation, the various methods of chemotherapy, radiation or phototherapy (50), anti-angiogenic drugs (51–53) and the “natural killer” (NK) cell-based immunotherapy (54) are available. However, all of these methods are intrinsic to the fact that the therapy of tumorigenesis, depending on the context, leads to resistance to these forms of treatment and ultimately to metastasis, and the patient is deprived of long-term good prognosis of both his total and disease-free survival rates.

While it is true that mutagenesis is firstly causing cancer via the alteration of the genetic material by mutagenic substances or radiation, this alteration is sufficient to cause cancer only if the mitochondria—the power organelles of the cell—are damaged. The knockdown or knockout of autophagy-related genes such as Atg5 and Atg7 ensuring mitochondrial fitness by removing defective mitochondria through mitophagy impairs this special form of autophagocytosis (37, 55–57).

Reactive oxygen species, called ROS for short, are free oxygen radicals, which, by their release, cause damage to the molecules essential for the functions of the cell, such as DNA, RNA, and a multiplicity of proteins and lipids. The accumulation of the oncometabolites succinate, fumarate and/or 2-hydroxyglutarate [which inhibit α-ketoglutarate (α-KG)—dependent enzymes, like the Jumonji domain-containing (JMJ), histone lysine demethylases (JHLDM), and ten-eleven translocation (TET) methylcytosine dioxygenases regulating both gene expression at the epigenetic level and the expression of oncogenic transcriptional programs] blocks terminal differentiation (8, 58–62).

Germline or somatic mutations cause the accumulation of these oncometabolites. Corresponding to it, the succinate dehydrogenase complex iron-sulfur subunit B (SDHB), the fumarate hydratase (FH), and the cytosolic isocitrate dehydrogenase [NADP^(+)^] 1 (IDH1), or the mitochondrial isocitrate dehydrogenase [NAD^(+)^] 2 (IDH2), are involved [(16); Figure 2Q]. A loss-of-function (LOF) mutation in SDHB and FH or gain-of-function (GOF) mutation in IDH1 and IDH2 lead to an increase in the cytosolic concentrations of succinate and fumarate on the one hand and 2-hydroxyglutarate on the other hand, respectively (61). Fumarate and succinate activate Kelch-like ECH-associated protein 1 (KEAP1) through a non-enzymatic post-translational protein modification called “succinylation,” which in turn activates the oncogenic transcription factor—namely the erythroid-derived nuclear factor 2 (NRF2 or NFE2) (63). In contrast, 2-hydroxyglutarate modulates the α-ketoglutarate-dependent activity of prolyl hydroxylases 1 and 2 (PHD1, 2) and the following hypoxia-inducible factor subunit 1α (HIF-1α) stabilization (Figure 2R), thus promoting the transformation of the developing malignancy (7, 8). Expression levels of transketolase (TKT)—an antagonist of α-KG—could, for instance, regulate the metabolic switch through HIF-1α and PDH2 via the α-KG-dependent dioxygenase signaling and the transcription of SDH and FH to control breast cancer metastasis (64). The concomitant oncogene signaling promotes the mitogen-activated protein kinase (MAPK) cascade (65), epidermal growth factor receptor (EGFR) signaling (66), and enhanced protection against the oncogene-driven mitochondrial outer membrane permeabilization (MOMP), mitochondrial permeability transition (MPT), senescence, and the regulated cell death (RCD) (67–70).

The epithelial-mesenchymal transition (EMT) is also dependent on down-regulation of succinate dehydrogenase and the subsequent accumulation of succinate in breast cancer progression and represents SDH as a potential key regulator of EMT [(71); Figure 2Q]. Down-regulation of succinate dehydrogenase B (SDHB) is common in central nervous system (CNV)—hemangioblastoma (72). Isocitrate dehydrogenase 1 (IDH1) (Figure 2Q)—mutant human gliomas show a high dependence of lactate and glutamate because of the resulting deficit in α-ketoglutarate (α-KG). The accompanying neuronal cells and astrocytes in the microenvironment are supplying them with these nutrients to replenish the TCA cycle (73).

As mentioned in the “Metabolism in Cancer” section of this review, tumor progression is associated with the rewiring of cancer metabolism. In addition to increased glycolysis and the increased use of PPP under hypoxic conditions, as they also occur in cells with mitochondrial defects, there is a realignment to other metabolic pathways. This realignment includes reductive glutamine metabolism (Figures 1A,B, 2I)—the main source of cytosolic citrate [(26, 74, 75); Figure 2L]. Serine metabolism (Figure 2D) is also important for lipid synthesis and the maintenance of redox homeostasis through the production of reducing equivalents, responsible for as much as 50% of cellular NADPH production (29, 76). This occurs via the mitochondrial serine hydroxymethyltransferase 2 (SHMT2) and the cytosolic SHMT1, which synthesize from tetrahydrofolate (THF) and glycine 5,10-methenyl-THF and further via the 5,10-methylene-THF dehydrogenase 1 and 2(L) (MTHFD1/2/2L), 5,10-methylene-THF (also called one-carbon or folate cycle). A set of reductive-oxidative conversions leads to a variety of THF subtypes that are required for purines, thymidine, and S-adenosylmethionine (SAM) biosynthesis (77). The latter is an important substrate for gene-regulatory methylation reactions.

Via the reversible oxidative decarboxylation of (S)-malate to pyruvate [(S)-malate + NAD(P)^+^ ↔ pyruvate + CO_2_ + NAD(P)H], performed by means of the cytosolic malic enzyme 1 (ME1)—also called malate dehydrogenase—which combines glycolysis with the TCA cycle, NAD(P)H is also obtained in PDACs and highly proliferating breast cancer [(27, 78); Figure 2S]. Cancer cells take up extracellular citrate from the blood via the plasma membrane citrate-transporting protein (pmCiC) that supports both the cancer cell metabolism and the proliferation of cancer cells through both its delivery to the glutamate and TCA metabolism or for fatty acid synthesis [(79); Figure 2C]. Similarly, acetoacetate, which is derived from acetyl-CoA, enhances oncogenesis through elevating the activity of BRAF kinase, which results in increased MAPK signaling (80, 81).

Furthermore, slightly increased ROS levels stimulate proliferation by inactivating tumor suppressor proteins such as the phosphatase and tensin homolog PTEN or stabilizing HIF1α [(82, 83); Figure 2T]. They manage and control mitochondrial biogenesis and its metabolism (84, 85). These slightly increased ROS levels arise, for example, as a side effect through the over-expression of ATPase inhibitory factor 1 (ATPIF1) and the resulting limitation of ATP production after the dimerization of the ETC complex V in the inner mitochondrial membrane (86, 87). However, even when ROS-derived senescence occurs, this may paradoxically result in increased proliferation via cell-extrinsic secretion of mitogenic factors to neighboring cells (88, 89).

In the development of the resistance of the cancer cell into regulated cell death (RCD), the mitochondrial transmembrane potential (Δψm) and thus, glycolysis increase in some tumors (90). Increased levels of glutathione (GSH) prevent the oxidation and translocation of cytochrome C from the mitochondria into the cytoplasm, thus preventing MOMP and apoptosis (91). Anti-oxidative, reducing equivalents are delivered through glycolysis and reductive glutamine carboxylation [(76, 92, 93); Figures 2D,I].

Furthermore, due to the low ROS levels, hormesis—already formulated by Paracelsus—occurs, together with the promotion of autophagy, which is reminiscent of ischemic preconditioning (94–96). In addition to glycolysis, adequate ATP supply by the mitochondria ensures optimal Ca^2+^ homeostasis and limited mitochondrial permeability transition (MPT) (86, 97). The resulting glucose deprivation causes even a change of glycolysis to OxPhos and, through mitochondrial elongation and mitophagy, the removal of dysfunctional components thanks to both the inhibition of dynamin 1-like protein (DNM1L) and the creation of a mitochondrial network (98, 99).

The heterogeneity between the extents of the use of oxidative phosphorylation (OxPhos) in relation to glycolysis depends on the origin and localization of the primary tumors and their metastases. Chemo-resistance develops because of the ability of cancer cells to rewire their metabolism flexibly. Increased OxPhos causes, among other things, increased expression of class I MHC molecules on the outer membrane of the cancer cells and thereby a reduction in their natural killer (NK) cell-mediated lysis. Twisting the polarization of M1- to M2-macrophages, which occurs mainly under hypoxic conditions using OxPhos, supports tumorigenesis (100–104).

Lactate-activated tumor-associated macrophages (TAMs) increase chemokine (CC) ligand 5 (CCL5) expression via Notch 1 and Jagged 2 signaling. Its activated receptor CCR5, like its ligand, is stimulated by the transforming growth factor beta 1 (TGF-ß1), which leads to increased aerobic glycolysis via activated AMPK and promotes EMT in the breast cancer cells investigated (105). Due to the further increase in glycolysis, there is a positive feedback loop, which further activates the TAMs via the increased lactate secretion. Lactate dehydrogenase A (LDH-A) expression has influence on the tumor microenvironment through HIF signaling, and the immune response is modulated via expression levels of hexokinase 1 and 2 (HK 1 and 2) and VEGF secretion (106). The increased lactate is responsible for impaired T cell function via hypoxia-reduced microRNA 34a (miR-34a) expression in gastric cancer-associated tumor-infiltrating lymphocytes, too (107).

The non-metabolic functions of lactate, which can be converted into pyruvate as an energy source in tumor cells, remain unknown. Zhang et al. (108) describe a previously unknown histone modification called lactylation, which is derived from lactate. The authors show, by identifying 28 lactylation sites on human and mouse histones, that lactylation of histone lysine residues displays an epigenetic modification, thus directly stimulating chromatin gene transcription. Glycolysis and lactate production is induced by hypoxia and bacterial stress, thus stimulating histone lactylation. Stimulation of M1 macrophages by exposing them to bacteria initiates histone lactylation that is temporally performed differently than histone acetylation. Histone lactylation increases in a late phase of M1 macrophage polarization inducing homeostatic wound healing genes by an endogenous “lactate clock,” which provides the opportunity to understand lactate's functions in infections and cancer.

A recent study (109) found that tumor-associated regulatory T cells (Tregs, also named suppressor T cells), in contrast to conventional T cells (Tconv), enhanced fatty acid synthesis (FAS), and fatty acid oxidation (FAO) to compensate for the lack of nutrients in the tumor, and they accumulated in its microenvironment, protecting the latter from infiltration by conventional activated T cells.

Elevated levels of mitochondrial reactive oxygen species (ROS) promote cancer metastasis through induction of EMT through metabolic remodeling via increased fatty acid β-oxidation and MAPK cascades in cancer stem cells (110). Immature neutrophils of low-density (iLDNs) exhibit increased metabolic flexibility and global biogenetic capacity to activate mitochondrial ATP production. Among other things, the granulocyte colony-stimulating factor (G-CSF), secreted by breast cancer cells mobilizes the cells migrating into the liver and promotes breast cancer metastasis via NETosis. Cancer cells mainly use glutamate and proline in the glucose-deprived environment (111). The microRNA miR-143 controls memory T cell differentiation by reprogramming T cell metabolism in esophageal carcinoma patients via a reduction in cell apoptosis and pro-inflammatory cytokine secretion. Glucose transporter 1 (GLUT-1)—the decisive target gene—and indolamine-2,3-dioxygenase (IDO) and its crucial metabolite kynurenine constitute the upstream regulators of miR-143 in memory T cells and the reprogramming of the tumor microenvironment metabolism by GLUT-1 (112). On the other hand, contrary to the need for hexokinase 2 (HK2) for cancer progression or growth in various cancer models, T cells can withstand the loss of hexokinase 2. HK2 is the most highly regulated enzyme in cancer and activated T-cells, which suggests that HK2 could be a promising target for cancer therapy of T-ALL leukemia (113).

## The Influence of Tumor Microenvironment on Early Malignancy, Full-Blown Tumor, and Cancer Stem Cells

Diversification and functional interaction between the tumor microenvironment and the non-transformed environment occur due to the metabolic needs of the increasing phenotypic and metabolic plasticity of developing malignancies (114–119). Cancer stem cells (CSCs) with a self-renewing potential show a predominantly glycolytic metabolism and are responsible for the local progression and recurrence of the tumor (120–123). Nevertheless, there are also exceptions; for example, the CSC's of ovarian tumors show a metabolism based more on oxidative phosphorylation (124); and there are also metabolic differences within the individual subgroups of CSCs within a tumor (125, 126).

Cancer cells, such as prostate cancer cells, reprogram so-called tumor-associated fibroblasts (TAFs) in the direction of glycolysis. By lactate secretion into the tumor microenvironment, they receive themselves a lactate-induced oxidative metabolism; this model is referred to as the “reverse Warburg effect” (127–130).

Recently, studies have shown that there could be a new model to understand the Warburg effect, concerning cancer metabolism in breast cancer and lymphoma. This hypothesis says that epithelial tumor cells provoke aerobic glycolysis in adjacent fibroblasts of the stroma. These fibroblasts secrete energy metabolites from aerobic glycolysis, like lactate and pyruvate that are absorbed via the monocarboxylate transporter 1 (MCT-1) and consumed in the TCA cycle by the cancer cells, resulting in a proper energy metabolite flow through ATP generation via OxPhos, promoting a higher cancer cell proliferation (127, 131). Essentially, the tumor-associated fibroblasts would feed the cancer cells in a kind of host-parasite conjunction. This new model still goes convenient with Warburg's statement that tumors (consisting of the cancer and the stroma) are shifting their metabolism from oxidative phosphorylation to aerobic glycolysis. This is also in line with new studies that show that breast cancer and endometrial carcinoma cells still depend on mitochondrial oxidative metabolism. With a parasitism-like behavior, PDACs drive the TAFs toward autophagy and thus generate local alanine availability, which the cancer cells harness as a carbon source via extracellular uptake of proteins by the so-called macropinocytosis [(132, 133); Figure 2G]. However, macropinocytosis also recruits fatty acids (FA) for the oxidative FA metabolism (FAO) of local adipocytes (134–137). The genome-wide analysis has shown critical regulation for adipocyte-associated breast cancer through two microRNAs—miR-3184-5p and miR-181-3p—which were most up- and down-regulated, with their direct targets being forkhead-box protein 4 (FOXP4) and the peroxisome proliferator-activated receptor alpha (PPARα). *In vitro* co-culture of breast cancer cells with mature adipocytes has resulted in an increase in proliferation, migration, and invasion via the Notch-induced EMT pathway and the increased production of cytokines and chemokines. Diabetes mellitus also promotes breast cancer progression (138).

The metabolic competition for nutrients with deprived availability has, as already mentioned, also direct effects on the immune surveillance by immune effector cells—which show similar metabolic behavior as the highly proliferating cancer cells—and thus on the evasion of immune surveillance by tumorigenesis (139–141).

In addition to the metabolic “parasitism,” there also exists a seemingly “symbiotic” form of metabolism happening between cancer cells of hypoxic, with those of normoxic areas and glycolysis-driven lactate transporting into oxygen-well-exposed areas. These areas are able to metabolize the lactate via OxPhos and, in turn, to provide the hypoxic areas with energy and bicarbonate (${\text{HCO}}_{3}^{-}$) ions to balance their proton surplus in the hypoxic centerpiece of tumor growth via the so-called connexin (gap-junction protein) -composited “communicating” transitions (142–144).

Ultimately, active ${\text{HCO}}_{3}^{-}$ transport from normoxic cells regulates the pH_i_ of hypoxic cancer cells in the tumor core and supports lactic acid discharge and acid-base transport through chemical titration between the alkaline peripheral cells and the acidic central cells via connexin channels in junction-coupled tumors to maintain pH homeostasis. Thereby, the discharge of lactate into the normoxic regions of the edges of the tumor represents a strategy for avoiding the competition for glucose in a nutrient- and oxygen-deprived microenvironment.

## The Metabolism of Cancer Cell Metastasis

Crucial for the patient's survival prognosis is the question of the presence of metastasis, called metastatic seeding or even dissemination. After a certain time, the tumor hits the limits of its growth. Hypoxia and hypoglycemia are increasing inside the tumor core [(145); Figure 1C]. If the support from the tumor microenvironment and re-vascularization of the tumor through the genesis of new blunted blood vessels, together with the reprogramming of metabolism, reach their limits, the chances for further tumor growth would remain in the re-orientation of its phenotype to invade the bloodstream or lymphatic vessels. Its subsequent trans-endothelial escape from the primary site into new, distal body sites would guarantee its continued survival but ultimately kill the patient (146). These distal sites of secondary tumor development are essentially with nutrients and oxygen richly supplied areas, as such the lungs, the liver, the brain, bones, the omentum, and the lymph nodes, thus providing the developing metastasis with the ideal conditions for further survival.

A first and important step in the development of metastasis of the tumor is the alteration of its cell-specific phenotype from a differentiated epithelial phenotype with a clear differentiation into an apical (outer region, facing the skin, or cell lumen) and basal (inner region, connected via a basal membrane with the underlying tissue) side into a mesenchymal phenotype. This phenotype increasingly loses its epithelial features and its polarization and assumes a migratory phenotype capable of altering its position, dissolving the cell-cell contacts to penetrate the basal membrane and to reduce the expression of adhesion molecules like E-cadherin, the epithelial cell adhesion molecule EpCAM, and keratin-14. The expression levels of other molecules, such as vimentin, N-cadherin, or fibronectin, are upregulated. Once again, switching on genes from embryonic development hereby determines the phenotype of the migrating cancer cell.

The mechanism responsible for the so-called “metastatic cascade” gave it its name: the epithelial-mesenchymal transition or EMT for short. EMT results in increased migratory and invasive potential of the malignancy (147, 148). For example, fumarate, succinate, or 2-hydroxyglutarate are able to repress transcription of microRNAs that inhibit metastasis by repressing Ten-eleven translocation methylcytosine dioxygenase 1 (TET1) or Jumonji domain-containing histone demethylase (JHDM) (149, 150). The microRNA [(miRNA) for short; it mostly consists of 21- to 23-nucleotide long, highly conserved, and non-coding RNAs, which play an important role at the post-transcriptional level of gene regulation] regulation of many gene targets makes them a therapeutically interesting topic (151, 152). Rather than inhibiting a single neoplastic process, the concomitant or delayed staging of many processes is re-sensitizing the cancer cell to therapy. However, this sword of miRNA regulation is double-edged: the non-specific nature of miRNA regulation often leads to undesirable interactions with target genes that are not involved in the regulation of cancer proliferation and metastasis. Nonetheless, proteasome inhibitors do not fail to work. MiRNA-based therapeutics have now undergone Phase 1 of clinical trials (152).

Additionally, up-regulation of the genes of the peroxisome proliferator-activated receptor gamma co-activator-1 alpha (PPARγ co-activator 1α, also PGC-1α) in breast cancer (153), or silencing of the mitochondrial membrane protein FAM210B in ovarian cancer provide optimal utilization of mitochondrial biogenesis and oxidative phosphorylation that is implemented through the down-regulation of pyruvate dehydrogenase kinase isozyme 4 (PDK-4) and the increased utilization of pyruvate from glycolysis via TCA cycle [(154); Figure 2U]. This process is important for mitochondrial-oxidative metabolism at the “growth bud” of the tumor, which links to the formation of cytoskeletal changes necessary for increasing motility of the migrating and invading malignancies (155–157). PGC-1α is essential for TGF-ß and Neu/ErbB2-driven breast carcinoma onset and resistance to biguanides such as metformin. The interaction of PGC-1α with the ShcA adapter protein thereby raises metabolism and breast tumor glucose-dependence. However, impaired ShcA signaling increases glutamine dependence due to a reduction in PGC-1α levels, mitochondrial efficiency, and metabolic versatility of breast cancer (158–161).

Mitophagy defects increase metastatic dissemination via slightly elevated ROS levels (162–165). This activates several signaling cascades, such as the proto-oncogenic tyrosine kinase SRC and protein tyrosine kinase 2 beta (PTK2B) signaling, which are essential for metastasis (165, 166). However, too many ROS inhibit metastasis through oxidative stress, leading to senescence or regulated cell death (167–169). Depending on the anatomical origin of the metastatic lesions, there is significant heterogeneity in the differential use of oxidative phosphorylation concerning glycolysis (116, 170, 171). Mitochondria are therefore the linchpin of cell signaling and metabolism, which decide about the determination of tumorigenesis (59, 172–174).

Conversely, mitochondrial dysfunction can lead to reduced NADH turnover and an increase in the cytosolic NADH concentration. Through the reductive glutamine carboxylation and, subsequently, via the malate dehydrogenase of the malate-aspartate shuttle, the NAD redox state is restored, and the cytosolic glyceraldehyde-3-phosphate dehydrogenase (GAPDH) and glycolysis activity is enhanced, resulting in an increased ATP production associated with increased cell migration. Thus, the NADH shuttle combines cytosolic reductive glutamine carboxylation with glycolysis in mitochondrial-dysfunctional cells (175–177).

There is some evidence that breast cancer patients with metabolic pathologies, such as diabetes and obesity, are less responsive to therapies and have a higher overall mortality rate, with hyperglycemia appearing to be responsible for this. It is also possible that the excess leptin production leads to an up-regulation of the leptin receptor and subsequent stimulation of the JAK2/STAT3 or PI3K/AKT signaling pathways via the gene regulation of Foxc2, Twist2, Vim, Akt3, and Sox2 to a CSC/EMT phenotype determined in triple-negative breast cancer cells (178). Additionally, suppression of the pyruvate dehydrogenase (PDH) complex (Figure 2U) via oncogenic microRNA-27b can deregulate breast cancer growth by shifting the metabolism from oxidative phosphorylation toward glycolysis, thus negatively affecting patient survival (151). Casein kinase 2 (CK2) modulates the pyruvate kinase M isoforms 1/2 ratio in favor of pyruvate kinase M2 (PKM2) (Figure 2P), thereby triggering EMT in colon carcinoma cell lines by increasing glycolytic activity and LDHA-driven proliferation (179).

The mechanotransduction of signals from the surrounding stiffening tissue of the tumor also determines the extent of the glycolytic drift. The publication data from Park et al. (180) show that the extent of the glycolysis of transformed non-small cell lung cancer cells reacts to the stiffness of the actomyosin cytoskeleton and that the mechano-sensing machinery of the surrounding tissue controls the cell metabolism. Non-malignant cells can adapt their energy production to changing environments. Cancer cells, on the other hand, react to the constantly changing mechanical ambient pressure during tumor progression with a constantly high glycolytic rate in the cancer cells. The transfer of human bronchial epithelial cells from stiff to soft substrates leads to a glycolysis reduction through the breakdown of phosphofructokinase (PFK). This degradation is mediated by the disassembly of stress fibers releasing the E3-ubiquitin ligase tripartite motif (TRIM) containing protein 21 (TRIM21). The transformed cells of non-small cell lung cancer that, despite the changed environmental conditions, maintain high glycolysis via the increased PFK expression, down-regulate TRIM21 and sequester residual TRIM21 on a subset of stress fibers that are insensitive to substrate stiffness.

While the assumption, in the past, was, that the epithelial and mesenchymal phenotypes of metastasizing cancer would just show a rigid switch between them, either epithelial or mesenchymal, now we know that there are several intermediary stages in between. This depends on the metabolic and signaling conditions in the tumor microenvironment, the invasion location into the vascular network, the conditions found in the site of trans-endothelial migration (extravasation) out of it, and the conditions found in the metastatic destination site. The current needs of the cancer cells not only determines the transition to the mesenchymal phenotype, but also is responsible for the reversed directed development toward the epithelial phenotype, called mesenchymal-epithelial transition (MET). A recent study (181) investigated transitional states tending more to the epithelial or the mesenchymal characteristics and classified them, besides measuring the levels of expression of adhesion molecules and molecules of the mesenchymal state, based on the levels of CD51, CD61, and CD106, receptor proteins that indicate a mesenchymal cell state. It turned out that the majority of the metastasizing cells did not wholly de-differentiate themselves to the mesenchymal phenotype to metastasize and to finalize the transition in a distant organ site.

Many cancer cells of the primary tumor, called circulating tumor cells (CTCs), leave the circulatory, or lymphatic system at their extravasation site and they have to change their metabolic phenotype to survive in the new tumor microenvironment of the organ of their destination site; thereby, only few of them show the required metabolic characteristics to build metastatic lesions. Many of them remain in a “sleeping” mode, called dormant CTCs, which prevents anoikis—the programmed death of cells, which were losing their contact to the cell matrix. These cells are reactivated only when the survival conditions improve in the microenvironment of their choice.

Metabolism is critical for survival and stress adaptation, and one believes that CTCs possess an individual signature to fulfill these metabolic requirements. These so-called disseminated tumor cells (DTCs) have a certain “predilection” for a host organ that has a specific metabolic signature for colonization at a specific organ site, but this also offers a new Achilles' heel for cancer therapy. Some tumors metastasize to specific organ sites, like prostate cancer to bone or pancreatic carcinoma to liver (182, 183); on the contrary, cancers from breast and lung are not fussy. Recent evidence gives a hint that the flexible character of metabolism is determining, whether the metastatic process is successful and promotes metastasis in distant organ destinations (184).

## Therapeutic Implications of the Metabolic Flexibility of the Tumor and Its Cancer Stem Cells

Embryonic and early fetal growth mainly requires glycolysis and constitutive growth factors (for instance, IGF, mTOR) among predominant anaerobic environmental conditions (low O_2_ partial pressure) and without evolution of functional mitochondria (185, 186). When the child is born and with contact to the oxygen in the atmosphere, many challenges of the environment and its adaptation to it, the developmental program adjustments, and the terminal differentiation of these developmental programs; shaping of the lymphoid system, maturation of the cells of the immune system and their thymus and bone marrow settlement are necessary for mitochondrial efficiency and strong immunity. Therefore, there is a need for bioenergetic health and strongly working cell-mediated and humoral immunity to give protection to external threads after birth and throughout life (187, 188).

Aging and oxidative stress negatively affect an efficient immunity, mitochondrial health, and weakens the immune system; they are also responsible for hypoxia, resembling the states of anabolic fetal development and may be the reason for tumorigenesis (189).

The activation of constitutive embryonic growth factors of epithelial cells and their epithelial-mesenchymal transition (EMT) induce immunoreactive tissue loss. A disrupted pyruvate transport, the biosynthesis of proteins enriched with branched and aromatic amino acids, enhanced activity of Serin/Threoninkinase IRAK-M (190, 191) and other growth paths, like mTOR/PI3Ks, VEGF, enhanced glycolysis to address the increased demands for aberrant cancer cell growth under hypoxia, and the disruption of the inhibition of cell-cell contact, accompany these events (192–194).

Effective immunity in acute inflammatory conditions has the power to exterminate cancer and to revise or prevent the initial stages of immune dysfunction. However, it has been shown that chronic inflammation and an aged immune system reduced stability of the genome, changed the expression of the factors of the inflammatory and anti-inflammatory machinery, altered the mitochondrial and ribosomal fitness and functionality, and disturbed the acid-base balance, increasing the risk of carcinogenesis in tissues. Anti-cancer drugs, derived from identifying multiple mutant or damaged genes or their related growth enzymes form the basis for late-stage cancer therapy as “targeted,” “precision,” or “cancer” therapy or even called “personalized medicine.” Nonetheless, this sort of therapy not infrequently shows drug-related, undesirable effects that threaten the lives of the patients leading in worst case to deadly multi-organ failure (MOF) (195, 196).

Activation of constitutive trophoblast growth factors required for orderly placental embryonic and fetal growth and receptor proteins—like pyruvate kinases and their activators—induce the production of anabolic components of metabolism affecting aberrant cancer cell growth and metabolism to facilitate glycolysis under hypoxic conditions with dysfunctional mitochondria. These characteristics are similar to those of the early fetal growth. Embryonic growth factors expressions facilitate the ameliorated glycolysis, and effects, named after Louis Pasteur, or Herbert Grace Crabtree (glucose), inducing immune tolerance, and generating a high-energy state that facilitates cancer proliferation—conditions not beneficial for the survival of non-malignant cells (197–202).

The crux at the heart of the choice of cancer therapy is due to the particular cell context. Attempts have been made to study the proliferation, cell cycle, migration, and invasion of cancer cells and their derived cancer stem cells to combat autophagy, mitophagy, the bilateral interaction within the tumor microenvironment, and the acquisition of blood vessels and thus of nutrients and oxygen via neo-angiogenesis. The targeting of factors that cause tumorigenesis with various anti-neoplastic drugs to prevent the primary as well as secondary tumor from spreading further has, in the best case, reduced its size or killed the tumor completely. Thus, complete healing of the patient has been achieved depending on the origin of cancer cells, the degree of their aggressiveness, and their stage of development, their ability to form metastases or cancer stem cells, and their choice of their metastasis site.

In addition to therapy with anti-neoplastic agents and classical chemotherapy, including radiation therapy (50), treatment with anti-angiogenic drugs (51–53) and “natural killer” (NK)—based therapy (54), several metabolic pathways of mitochondria influence the therapeutic answer. For example, metabolic enzymes such as the mutated mitochondrial form of isocitrate dehydrogenase 2 (IDH2), which block terminal differentiation, have been harnessed for the development of drugs (58, 203, 204). BRAF^V600E^–inhibition by vemurafenib acts as a “switch” from glycolysis to oxidative phosphorylation and concomitant therapy resistance. The inhibitor of the electron transport chain honokiol reverses this resistance (205). Oncogenic ablation of KRAS^G12D^–driven PDAC selects a resistant population that relies on oxidative phosphorylation (206). Breast cancer cells, after MYC/KRAS- or MYC/ERBB2- ablation (207), are similar to glioma cells in their acquired resistance to phosphoinositol-3-kinase (PIK3) inhibitors (208).

The activities of many different ATP-binding cassette (ABC) family transporter proteins that mediate chemo-resistance against various cancer therapeutics through the export of xenobiotic, depend on OxPhos-driven ATP availability (209); and in some cases, a higher inflammatory state is driven by OxPhos, with interleukin 6 (IL-6) and tumor necrosis factor alpha (TNF-α) secreted in the microenvironment of the tumor (210). Therefore, cancer cells, which show higher glycolysis, develop a resistance to therapy through cells' intrinsic and extrinsic pathways following a “switch” to OxPhos. Furthermore, malignant cells that use pre-dominant oxidative phosphorylation for energy production, including the CSCs of pancreatic carcinoma, may develop resistance to ETC inhibition because they acquire MYC-dependent glycolytic metabolism (211). Chemo-resistant ovarian cancer shows a drift from OxPhos to glycolysis, followed by an enhanced production of the PPP-dependent NADPH antioxidant, which ensures redox homeostasis in the cytosol (212). Thus, chemo-resistance would be a result of the ability of cancer cells to shape their mitochondrial metabolism flexibly so that they can escape and survive constraints such as regulated cell death (RCD) that they would experience through therapy.

Chao et al. (213) used the targeting of lactic acidosis in the so-called TILA-TACE (targeting of intratumoral lactic acidosis-transarterial chemo-embolization) therapy of hepatocellular carcinomas (HCCs) larger than 5–10 cm and not suitable for curative therapy. First, they alkalized the tumor with a sodium bicarbonate solution via tumor-feeding vessels and subsequently, by embolization of these vessels, they cut the tumor off from a subsequent glucose supply. As they show in their study, the alkalization of the glucose-deprived tumor transfers the metabolism from the economy and efficiency mode back to the “Warburg” mode. After the embolization of the glucose-supplying vessels, the tumor metabolism literally runs “hot,” which ultimately leads to intratumoral apoptotic or necrotic cell death (214–216).

## Conclusion

In everything that we have learned so far about the development of cancer, there is an all-encompassing basic idea that successful therapy must always take into account the therapy evasion behavior caused by the manifold metabolic mechanisms of cancer cells we reported in this review. Due to the metabolic flexibility of the tumor cells, the therapeutic approach usually shifts the metabolism only in a different direction and often provokes a mechanism of resistance, mainly due to the ability of the mitochondria to alter the energy and metabolite synthesis to sustain an adequate proliferation, invasion, and migration capacity to continue tumorigenesis successfully. Therefore, it is important to include these tumor and stem cell mechanisms in the therapeutic approach, and to consider the simultaneous or staggered delivery of drugs or natural products that block multiple metabolic pathways of the cancer cell and at best, would lead to apoptotic cell death.

The “Warburg-like” effect promotes active proliferation in rapidly dividing embryonic tissues as well as in tumorigenesis of cancer. Because of this metabolic feature, the important diagnostic technique of ^18^F-fluorodeoxyglucose positron emission tomography (^18^FDG-PET) (217) was developed and since then used in the clinic for the diagnosis of malignant diseases. Momcilovic et al. (218) have been using 4-[^18^F] fluorobenzyl-triphenylphosphonium (^18^FBnTP)—a positively charged tracer-ion that is accumulated on the negatively charged inner membrane of mitochondria—to distinguish squamous cell carcinoma from adenocarcinoma in mice, thus providing the opportunity to target their different dependency on oxidative phosphorylation. ^18^FBnTP-PET, together with ^18^FDG-PET, provides us with a new tool for *in vivo*-imaging energy and metabolites fluxes to further widen up our diagnostic horizon in treating different states of developing cancers. We can thus safely claim that, almost 100 years ago, Otto Warburg laid the foundation for today's cancer diagnosis and therapy (219).

## Author Contributions

Conception and design of the manuscript was done by ML and CG. Proofreading was done by GE. All authors read and approved the final manuscript.

### Conflict of Interest

The authors declare that the research was conducted in the absence of any commercial or financial relationships that could be construed as a potential conflict of interest.
